# A cross-metathesis approach to novel pantothenamide derivatives

**DOI:** 10.3762/bjoc.12.95

**Published:** 2016-05-13

**Authors:** Jinming Guan, Matthew Hachey, Lekha Puri, Vanessa Howieson, Kevin J Saliba, Karine Auclair

**Affiliations:** 1 Department of Chemistry, McGill University, 801 Sherbrooke Street West, Montreal, H3A 0B8, Canada; 2 Research School of Biology, College of Medicine, Biology and Environment, The Australian National University, Canberra, Australian Capital Territory, 2601, Australia; 3 Medical School, College of Medicine, Biology and Environment, The Australian National University, Canberra, Australian Capital Territory, 2601, Australia

**Keywords:** antibiotic, antiplasmodial, coenzyme A, metathesis, pantothenate

## Abstract

Pantothenamides are known for their in vitro antimicrobial activity. Our group has previously reported a new stereoselective route to access derivatives modified at the geminal dimethyl moiety. This route however fails in the addition of large substituents. Here we report a new synthetic route that exploits the known allyl derivative, allowing for the installation of larger groups via cross-metathesis. The method was applied in the synthesis of a new pantothenamide with improved stability in human blood.

## Introduction

Bacteria, fungi, and parasites are all rapidly acquiring resistance to currently applied antimicrobials and as a result, our ability to treat infections effectively is diminishing. Efforts to control infections in this resistance era have taken a variety of paths from a renewed push for novel antimicrobial agents to a fresh understanding of antibiotic resistance mechanisms [1]. One successful research direction has been to revisit “older” unexploited structural classes of antimicrobials [2–5].

**Figure 1:**
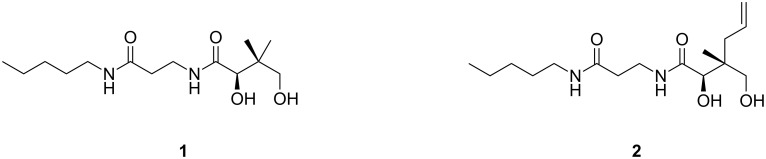
Chemical structures of *N*-pentylpantothenamide (**1**) and of its methyl allyl derivative **2**.

As first demonstrated in the 1970s [6], many pantothenamides show antimicrobial activity [7–12], including antibacterial, antifungal and/or antiplasmodial activity. For example, what has now become the benchmark pantothenamide, *N*-pentylpantothenamide (**1**, Figure 1), is active against *Escherichia coli* [6] and *Staphylococcus aureus* [13], at minimum inhibitory concentrations (MICs) in the low micromolar range. Pantothenamides are however rapidly degraded by enzymes, the pantetheinases, that normally hydrolyze pantetheine to pantothenate and cysteamine in human blood [10], which precludes their clinical application.

The antimicrobial activity of pantothenamides is believed to arise from at least two mechanisms depending on the microbial strain. In *E. coli* pantothenamides may mimic pantetheine and are extended into a thiol-lacking coenzyme A (CoA) derivative by some of the natural CoA biosynthetic enzymes (pantothenate kinase or PanK, phosphopantetheine adenylyltransferase and dephosphocoenzyme A kinase). The resulting inactive CoA analog affects downstream pathways such as the acyl carrier protein activation required for fatty acid biosynthesis [13–16]. In *S. aureus*, however, recent studies suggest that the antimicrobial activity correlates better with an inhibition of PanK, the regulatory enzyme in CoA biosynthesis in several organisms [17].

Additional studies on pantothenamides are thus easily justified based on their different structural scaffold and new mechanism of action compared to the antimicrobials currently in use.

The vast majority of reported pantothenamide derivatives are modified at the amine moiety [7,9–10]. Among the rare other variations, derivatives with different carbon-chain lengths in the β-alanine moiety have recently been reported by de Villiers et al. [12]. Interestingly, some of these show improved stability in the presence of blood pantetheinases. Modifications at the geminal dimethyl moiety of pantothenamides have also proven quite successful [11,18]. One of the promising derivatives reported is a methyl allyl (**2**, Figure 1) with a MIC of 3.2 μM against *S. aureus* and MRSA, compared to MICs of 7 μM for **1** against the same strains [18]. This result encouraged us to synthesize more derivatives with different substituents replacing the geminal dimethyl moiety. Although quite versatile and stereoselective, the reported synthetic methodology to access such analogs decreases in efficiency with increasing size of the group used to replace the methyl residue [11] . We report here a synthetic method to access larger substituents at the geminal dimethyl group. This route takes advantage of the reported path to **2** combined with cross-metathesis.

## Results and Discussion

### Establishing the synthetic route

**Figure 2:**
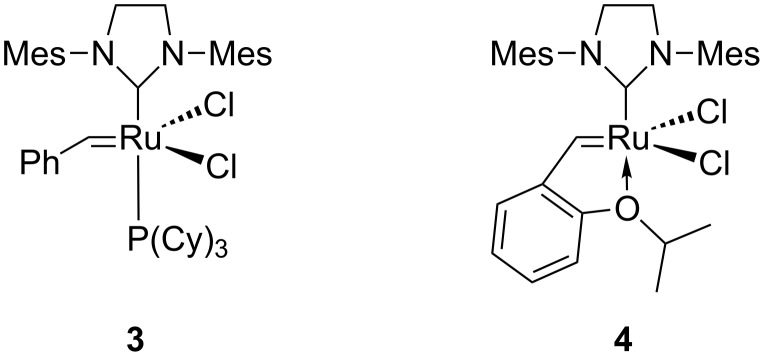
Structure of Grubbs’ catalysts used in this study.

**Scheme 1:**
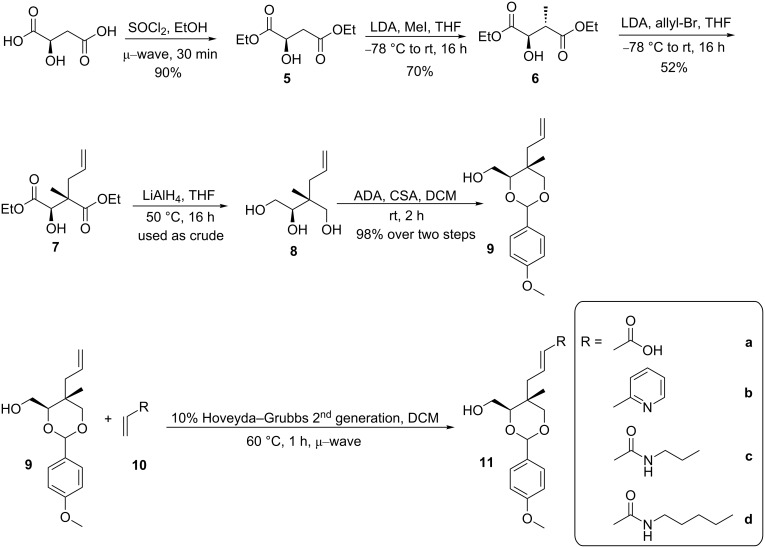
From (*R*)-malic acid to allyl derivatives **8** and **9** tested in cross-metathesis. Details are provided in the main text. μ-wave: microwave.

Based on the success of the synthetic route published by Akinnusi et al. [18] to generate derivatives modified at the geminal dimethyl moiety of pantothenamides, it was envisaged that allyl derivatives, such as **2** or its precursors, could be good starting points to add larger moieties via cross-metathesis [19]. Two cross-metathesis catalysts were used here (Figure 2): Grubbs’ 2^nd^ generation catalyst (**3**) and the more versatile Hoveyda–Grubbs’ 2^nd^ generation catalyst (**4**). Compounds **8** and **9** were chosen for initial tests with this reaction (Scheme 1).

The synthesis of compounds **8** and **9** was carried out as previously described (Scheme 1) except for some improvements in the esterification step [18]. Akinnusi et al. reported using reflux conditions (100 °C) with ethanol and thionyl chloride added in two portions separated by three hours [18]. Alternatively, it was found here that a microwave reactor reduced the overall reaction time down to 30 minutes, with an isolated yield of 90%, comparable to that of the thermal reaction.

### Metathesis reaction optimization

In 2003, Grubbs published a classification system for cross-metathesis catalysts and substrates, defining the substrates by type ranging from I to IV depending on their level of homodimerization observed in a metathesis reaction [20]. Type I substrates are defined as resulting in fast homodimerization under the conditions of the reaction. Such substrates include terminal alkenes of low steric bulk. Type II and type III substrates, with the latter being bulkier, show slow or no detectable homodimerization, respectively. Type IV is a separate class, including substrates that are spectators to metathesis yet do not inactivate the catalyst. It has been shown that for optimal results (minimal homodimerization) the two olefins to undergo cross-metathesis should be of different types [20].

**Scheme 2:**
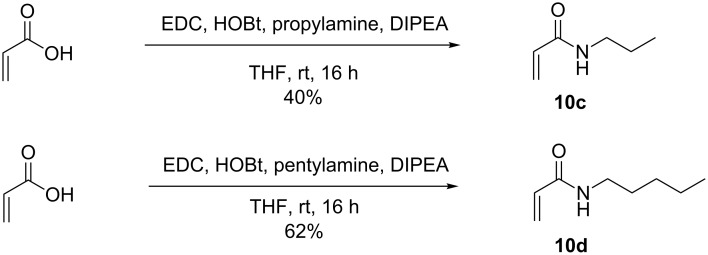
Synthesis of compounds **10c** and **10d**.

The two intermediates selected as possible reactants, compounds **8** and **9**, were expected to present type II and III behavior, respectively. Acrylic acid (**10a**) is known to react well in metathesis with catalyst **3** and therefore was used to test the suitability of compounds **8** and **9** in metathesis at 40 °C overnight [21]. As expected based on its large number of accessible conformations, no metathesis product was observed with compound **8** when reacted with acrylic acid. Compound **9** however was more promising and demonstrated type III substrate characteristics (no homodimerization observed) when used with the Grubbs’ 2^nd^ generation catalyst (**3**) [20]. The presence of a *p-*methoxybenzaldehyde acetal protecting group in **9** is believed to tie back the alcohols and prevent them from coordinating and deactivating the ruthenium catalyst. When testing the scope of the metathesis reaction with **9**, a variety of partners were chosen, including not only acrylic acid (**10a**), but also 2-vinylpyridine (**10b**), propylacrylamide (**10c**), and pentylacrylamide (**10d**, Table 1). Based on the classification reported by Grubbs [20], these substrates are type II when used in the presence of catalyst **3**. Propyl- and pentylacrylamide (**10c** and **10d**) were synthesized from acrylic acid and the respective primary amines as shown in Scheme 2.

**Table 1:** Optimization of the cross-metathesis reaction between **9** and various alkenes.

Cross-metathesis partners	Conditions used	Isolated yield (%) using catalyst **3**	Isolated yield (%) using catalyst **4**

**10a**	DCM, thermal 40 °C, 16 h, 5 mol % catalyst	50	N/A^a^
**10b**	DCM, thermal 40 °C, 16 h, 10 mol % catalyst	14	N/A
	DCM, μ-wave^b^ 60 °C, 60 min, 10 mol % catalyst	N/A	67
**10c**	DCM, thermal 40 °C, 16 h, 10 mol % catalyst	25	30
	DCM, μ-wave 60 °C, 60 min, 10 mol % catalyst	N/A	30
**10d**	DCM, thermal 40 °C, 16 h, 10 mol % catalyst	30	40
	DCM, μ-wave 60 °C, 60 min, 10 mol % catalyst	N/A	60

^a^N/A: not applicable; ^b^μ-wave: microwave.

Although a reasonable yield (50%) was obtained at the first attempt of reacting **9** with acrylic acid (**10a**) in the presence of catalyst **3** (Table 1), the reaction with pentylacrylamide (**10d**) proved less successful (30%), yet superior to that of propylacrylamide (**10c**, 25%). These low yields might be explained by homodimerization of the acrylamide moiety. 2-Vinylpyridine (**10b**) reacted in even lower yields (14%), as expected based on the known deactivation of catalyst **3** in the presence of pyridinyl ligands [22].

A previous work by Grubbs and co-workers has demonstrated that after dissociation from the metal complex, phosphine ligands can attack the carbine [23]. Thus a phosphine-free catalyst was desirable, and the Hoveyda–Grubbs’ 2^nd^ generation catalyst (**4**) was chosen. As predicted, the yields increased (Table 1) when catalyst **4** was used for the reaction of **9** with propylacrylamide (**10c**) and pentylacrylamide (**10d**). The *trans*-isomer was the only one detected by ^1^H NMR of the crude reaction mixture in all cases.

In the hope of further improving the yield of the reaction, a microwave reactor was employed. Indeed, a number of literature reports have demonstrated that microwave-assisted cross-metathesis reactions were complete in a fraction of the time required for the thermal process and provided a cleaner reaction [24–27]. In the cases of 2-vinylpyridine (**10b**) and pentylacrylamide (**10d**), not only did the reaction mixture appear cleaner by TLC, but a dramatic increase in yield (67% and 60%, respectively) was also observed when they were reacted with **9** in the presence of catalyst **4** at 60 °C for 60 minutes.

### Synthesis of a novel pantothenamide derivative

**Scheme 3:**
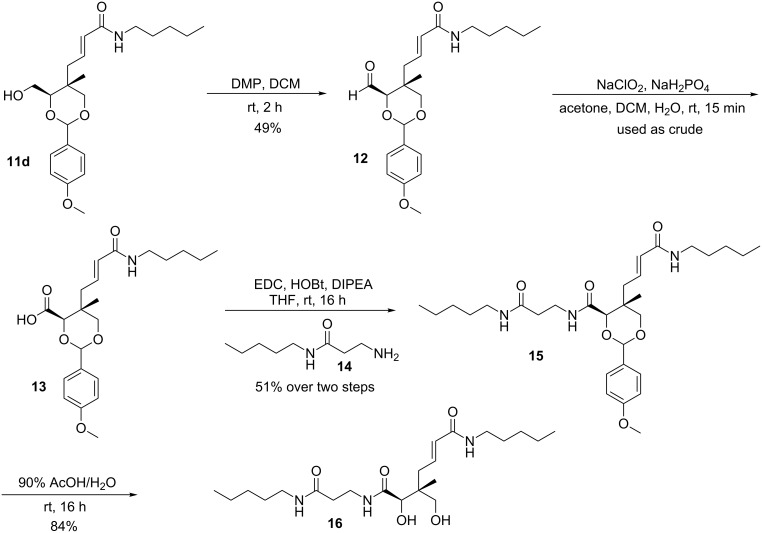
Synthesis of **16**.

With the successful optimization of the metathesis step, pentylacrylamide derivative **11d** was chosen as the molecule to be extended to pantothenamide **16**. As outlined in Scheme 3, **16** was assembled from **11d** by first oxidation to the corresponding aldehyde **12** using Dess–Martin periodinane in wet DCM. Further oxidation to the carboxylic acid **13** was achieved using mild chlorite oxidation. The product was used in the next reaction without further purification due to its low stability.

The required amine **14** to be coupled to **13** was synthesized from Boc-protected β-alanine and pentylamine using a method reported by Hoegl et al. [11]. Deprotection of the Boc group with trifluoroacetic acid (TFA) provided the free amine **14**, which was directly coupled to the carboxylic acid to yield **15**. Finally, the desired compound **16** was obtained after acid deprotection of the *p*-methoxybenzaldehyde acetal in a 90% aqueous acetic acid solution (84% yield).

### Biological studies

**Figure 3:**
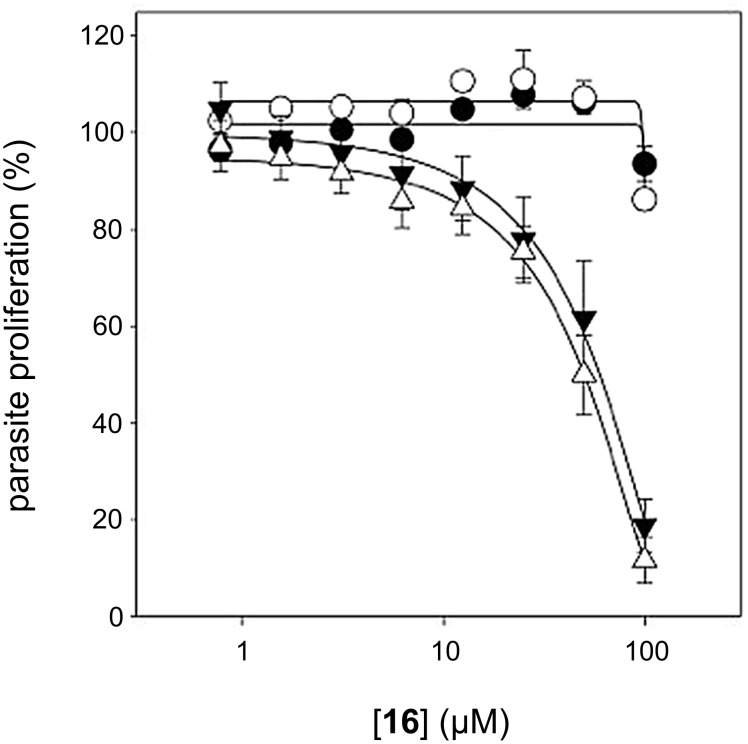
In vitro antiplasmodial activity of compound **16** in growth medium containing pantetheinase (open symbols) or in medium in which the pantetheinase had been inactivated (dark symbols). The antiplasmodial activity of this compound can be antagonized by increasing the extracellular concentration of pantothenate from 1 µM (triangles) to 100 μM (circles).

The antimicrobial activity of **16** was investigated. No visible growth inhibition was observed for *E. coli* or *S. aureus* in the presence of compound **16** (up to 512 μM). To determine if the lack of activity was due to poor target affinity or cell-permeability issues, kinetic studies with purified *E. coli* PanK were performed as previously reported [28]. Compound **16** was neither a good substrate nor an inhibitor of this enzyme, hence explaining its lack of antibacterial activity towards *E. coli*. On the other hand, compound **16** was found to inhibit the growth of *Plasmodium falciparum* with an IC_50_ value of 60 ± 11 μM (*n* = 3) in the absence of pantetheinase (Figure 3). Interestingly, its IC_50_ was unaffected by the presence of pantetheinase (51 μM ± 7, *n* = 6), confirming that modification of pantothenamides at the geminal dimethyl moiety is a viable strategy to overcome the blood stability issues that plague this family of compounds [11]. In order to verify if **16** was targeting CoA biosynthesis as reported for other pantothenamides, the IC_50_ was also measured in the presence of excess pantothenate (100 µM), in the presence or absence of pantetheinase. The dramatic loss of activity observed confirms that compound **16** acts on the CoA biosynthesis/utilization pathway.

## Conclusion

In summary, we have successfully developed a new synthetic route that exploits the known allyl derivative **2**, allowing for the installation of larger groups via cross-metathesis. Considering the importance of pantothenamides as a potential new class of antimicrobial agents, and the higher stability observed for **16** in blood, we expect this stereoselective synthetic route to find utility in accessing other new pantothenamide derivatives.

## Supporting Information

File 1 Experimental data.

File 2 NMR spectra.
