# Supplementary material for: A cross-metathesis approach to novel pantothenamide derivatives
Source: Beilstein J Org Chem. 2016 May 13;12:963–8. doi: 10.3762/bjoc.12.95 (PMC4902030; doi:10.3762/bjoc.12.95)
Supplement: File 1 — Experimental data. [file Beilstein_J_Org_Chem-12-963-s001.pdf]

Supporting Information File 1

for

A cross-metathesis approach to novel pantothenamide derivatives

Jinming Guan[1], Matthew Hachey[1], Lekha Puri[1], Vanessa Howieson[2], Kevin J. Saliba[2,3] and Karine Auclair*[1]

Address: [1]Department of Chemistry, McGill University, 801 Sherbrooke Street West, Montreal, H3A 0B8, Canada, [2]Research School of Biology, College of Medicine, Biology and Environment, The Australian National University, Canberra, Australian Capital Territory, 2601, Australia and [3]Medical School, College of Medicine, Biology and Environment, The Australian National University, Canberra, Australian Capital Territory, 2601, Australia

Email: Karine Auclair - karine.auclair@mcgill.ca

* Corresponding author

Experimental data

Table of Contents

1. Chemistry S2
2. Biological studies...S12
3. References S13

1. Chemistry

1.1. General information

Unless otherwise mentioned, all reagents and buffer components were purchased from Sigma-Aldrich Canada Ltd. (Oakville, ON, Canada). Reagents and solvents were used without further purification unless otherwise stated. Dry solvents were obtained from an Innovative Tech Pure Solve MD-7 Solvent purification system. Flash chromatography and TLC analysis (F-254) were performed with 60 Å silica gel from Silicycle (Quebec, QC, Canada). HRMS spectra were acquired at the McGill University Mass Spectral Facility by ESI on an EXACTIVE instrument in orbitrap mode. LRMS was performed using a Finnigan LCQDUO mass spectrometer with ESI without fragmentation. Microwave reactions were performed using a Biotage microwave system.

1.2. Synthesis

1.2.1. Diethyl (*R*)-2-hydroxysuccinate (5)

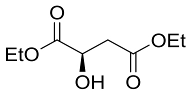

Compound **5** was prepared as previously reported [1] or using the microwave conditions as described below. (*R*)-Malic acid (1.0 g, 7.5 mmol) was weighed into a microwave tube equipped with a stir bar before addition of anhydrous ethanol (5 mL). Thionyl chloride (0.25 mL, 3.4 mmol) was next added dropwise, and the tube was sealed. The resulting light yellow mixture was allowed to react in a microwave reactor for 30 min at 100 °C. Then the yellow reaction mixture was diluted in diethyl ether (40 mL) and

washed with a saturated solution of $NaHCO_3$ (20 mL). The organic layer was dried over anhydrous Na_2SO_4. The white solid was filtered off and the solution concentrated in vacuo by rotary evaporation. The crude product was obtained as a clear, light-yellow oil, which was used directly in the next step. Yield: 1.3 g, 90%. Characterization matched previous report of this known compound [1].

1.2.2. (2*R*,3*S*)-Diethyl 2-hydroxy-3-methylsuccinate (6)

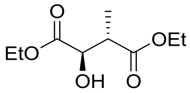

Compound **6** was prepared as previously reported in 70% yield. Characterization matched previous report of this known compound [1,2].

1.2.3. (2*S*,3*R*)-Diethyl 2-allyl-3-hydroxy-2-methylsuccinate (7)

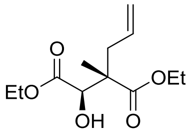

Compound **7** was prepared from **6** as previously reported in 52% yield. Characterization matched previous report of this known compound [1].

1.2.4. ((4*R*,5*R*)-5-Allyl-2-(4-methoxyphenyl)-5-methyl-1,3-dioxan-4-yl)methanol (9)

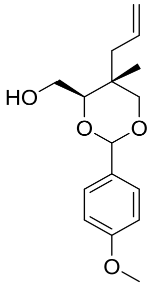

Compound **9** was prepared from **7** via **8**, as previously reported [1], in 98% yield over two steps. Characterization matched previous report of this known compound [1].

1.2.5. General procedure 1 for acrylamide coupling

EDC (4.2 mmol) and HOBt (5.0 mmol) were dissolved in dry THF (10 mL). Acrylic acid (100 mg, 1.4 mmol) was added, followed by the desired amine (2.1 mmol). Diisopropylethylamine (14 mmol) was added and the reaction was stirred for 16 h at rt. The mixture was then poured into a separatory funnel containing saturated NH_4Cl solution (20 mL), and extracted in ethyl acetate (3 × 40 mL). The organic layers were dried over anhydrous Na_2SO_4, the solid filtered off, and the solution concentrated in vacuo by rotary evaporation. Flash chromatography using a gradient of 0–100% ethyl acetate in hexanes was used to purify the desired compounds.

1.2.5.1. *N*-Propylacrylamide (10c)

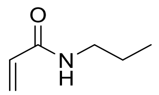

Prepared using general procedure 1 for acrylamide coupling. Characterization matched previous report of this known compound [3]. Yield: 57 mg, 40%. R_f = 0.3 (100% ethyl acetate).

1.2.5.2. *N*-Pentylacrylamide (10d)

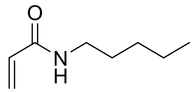

Prepared using the general procedure 1 for acrylamide coupling. This compound is claimed in a patent [4]. Yield: 121 mg, 62%. R_f = 0.24 (ethyl acetate/hexanes, 3:7). ^{1}H NMR ($CDCl_3$, 300 MHz) δ 6.26 (dd, J = 17.0, 1.7 Hz, 1H), 6.08 (dd, J = 17.0, 10.1 Hz, 1H), 5.74 (s, 1H), 5.61 (dd, J = 10.1, 1.7Hz, 1H), 3.31 (m, 2H), 1.59-1.45 (m, 2H), 1.41-1.21 (m, 4H), 0.88 (t, J = 6.9 Hz, 3H); ^{13}C NMR ($CDCl_3$, 125 MHz) δ 165.5, 131.0, 126.1, 39.6, 29.3, 29.1, 22.4, 14.0; HRMS for $C_8H_{15}NO$ $[M + H]^+$ calcd. 142.1226, found 142.1225.

1.2.6. (*E*)-4-((4*R*,5*R*)-4-(Hydroxymethyl)-2-(4-methoxyphenyl)-5-methyl-1,3-dioxan-5-yl)but-2-enoic acid (11a)

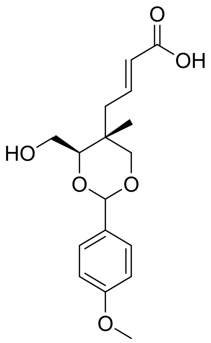

Grubbs' 2nd generation catalyst **3** (12 mg, 5 mol %) was weighed into a flask in a N_2 atmosphere glovebox. The flask was capped, removed from the glovebox, and placed under a N_2 line. To this flask compound **9** (78 mg, 0.28 mmol) dissolved in dry DCM (4 mL) was added, followed by acrylic acid (20 mg, 0.28 mmol). The flask was attached to a reflux condenser and the reaction was stirred at 40 °C for 16 h under N_2. Flash chromatography using a gradient of 0–100% ethyl acetate in hexanes was used to purify the desired compound, which was a brownish oil after evaporation of all solvents. Yield: 47 mg, 50%. R_f = 0.3 (100% ethyl acetate). ^{1}H NMR ($CDCl_3$, 400 MHz) δ 7.42 (d,

J = 8.7 Hz, 2H), 7.03 (dt, *J* = 15.6, 8.0 Hz, 1H), 6.91 (d, *J* = 8.7 Hz, 2H), 5.91 (d, *J* = 15.6 Hz, 1H), 5.46 (s, 1H), 3.81 (s, 3H), 3.75 (m, 5H), 2.24-2.15 (m, 2H), 1.22 (s, 3H); ESI-MS for $C_{17}H_{22}O_6$ $[M+Na]^+$ calcd. 345.14, found 345.16.

1.2.7. General procedure 2 for metathesis reactions in the microwave reactor

Hoveyda–Grubbs' 2nd generation catalyst **4** (22 mg, 10 mol %) was weighed into a vial in a N_2 atmosphere glovebox. The vial was capped, removed from the glovebox, and placed under a N_2 line. To a microwave vial, compound **9** (100 mg, 0.36 mmol), dissolved in dry, degassed DCM (0.5 mL), was added. The desired coupling partner (0.18 mmol), which was dissolved in DCM (0.5 mL), was next added. The catalyst dissolved in DCM (1 mL) was finally added. The vial was then purged with N_2 and sealed. The reaction was run for 60 min at 60 °C in a microwave reactor. Flash chromatography using a gradient of 0–100% ethyl acetate in hexanes was used to purify the desired compounds.

1.2.7.1. ((4*R*,5*R*)-2-(4-Methoxyphenyl)-5-methyl-5-((*E*)-3-(pyridin-2-yl)allyl)-1,3-dioxan-4-yl)methanol (11b)

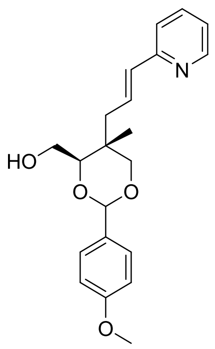

Prepared using the general procedure 2 under microwave irradiation from compound **9** (20 mg, 0.080 mmol). Yield: 17 mg, 67%. R_f = 0.35 (100% ethyl acetate). ^{1}H NMR ($CDCl_3$, 500 MHz) δ 8.57 (d, *J* = 4.5 Hz, 1H), 7.76 (t, *J* = 7.5 Hz, 1H), 7.42 (d, *J* = 8.7 Hz, 2H), 7.33 (d, *J* = 7.8 Hz, 1H), 7.25-7.22 (m, 1H), 6.91-6.87 (m, 3H), 6.57 (d, *J* = 15.6 Hz, 1H), 5.46 (s, 1H), 3.90 (dd, *J* = 8.6, 2.9 Hz, 1H), 3.84-3.80 (m, 3H), 3.80 (s, 3H), 3.74-3.69 (m, 1H), 2.30-2.23 (m, 2H, H-8), 1.25 (s, 3H, H-7); ESI-MS for $C_{20}H_{23}NO_4$ $[M+Na]^+$ calcd. 364.16, found 364.24.

1.2.7.2. (*E*)-4-((4*R*,5*R*)-4-(Hydroxymethyl)-2-(4-methoxyphenyl)-5-methyl-1,3-dioxan-5-yl)-*N*-propylbut-2-enamide (11c)

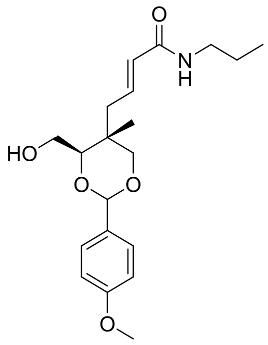

Prepared using the general procedure 2 under microwave irradiation from compound **9** (20 mg, 0.080 mmol). Yield: 8.0 mg, 30%. R_f = 0.2 (100% ethyl acetate). ^{1}H NMR ($CDCl_3$, 400 MHz) δ 7.42 (d, *J* = 8.7 Hz, 2H), 6.90 (d, *J* = 8.7 Hz, 2H), 6.82 (dt, *J* = 15.3, 7.9 Hz, 1H), 5.81 (d, *J* = 15.3 Hz, 1H), 5.44 (s, 1H), 3.95-3.38 (m, 8H), 3.30 (dt, *J* = 13.4, 6.8 Hz, 2H), 2.16-2.10 (m, 2H), 1.62-1.49 (m, 2H), 1.20 (s, 3H), 0.94 (t, *J* = 7.4 Hz, 3H); HRMS for $C_{20}H_{29}NO_5$ $[M-1]^-$ calcd. 362.1973, found 362.1976.

1.2.7.3. (*E*)-4-((4*R*,5*R*)-4-(Hydroxymethyl)-2-(4-methoxyphenyl)-5-methyl-1,3-dioxan-5-yl)-*N*-pentylbut-2-enamide (11d)

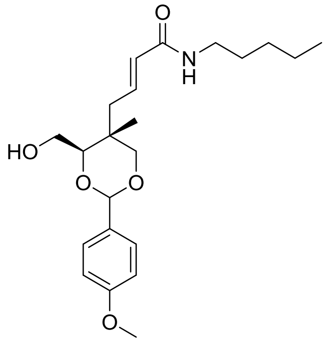

Prepared using the general procedure 2 under microwave irradiation from compound **9** (110mg, 0.40 mmol). Yield: 46 mg, 60%. R_f = 0.4 (100% ethyl acetate). ^{1}H NMR ($CDCl_3$, 500 MHz) δ 7.42 (d, *J* = 8.7 Hz, 2H), 6.90 (d, *J* = 8.7 Hz, 2H), 6.81 (dt, *J* = 15.5, 7.9 Hz, 1H), 5.80 (d, *J* = 15.0 Hz, 1H), 5.44 (s, 2H), 3.82-3.64 (m, 8H), 3.32 (m, 2H), 2.19-2.07 (m, 2H), 1.57-1.49 (m, 2H), 1.36-1.29 (m, 4H), 1.20 (s, 3H), 0.90 (t, *J* = 6.9 Hz, 3H); ^{13}C NMR ($CDCl_3$, 125 MHz) δ 164.9, 160.2, 138.1, 130.6, 127.6, 127.2, 113.7, 101.9, 84.3, 76.7, 61.4, 55.3, 39.6, 38.4, 34.9, 29.3, 29.1, 22.4, 17.7, 14.0; HRMS for $C_{22}H_{33}NO_5$ $[M+Na]^+$ calcd. 414.2251, found 414.2264.

1.2.8. (*E*)-4-((4*R*,5*R*)-4-Formyl-2-(4-methoxyphenyl)-5-methyl-1,3-dioxan-5-yl)-*N*-pentylbut-2-enamide (12)

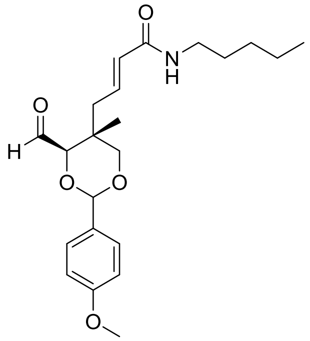

To compound **11d** (240 mg, 0.61mmol) in DCM (2 mL) was added Dess–Martin periodinane (520 mg, 1.23 mmol). The mixture was stirred at rt for 2 h. The mixture was diluted in diethyl ether (10 mL) and washed with a $Na_2S_2O_3/NaHCO_3$ solution (4.5 g $Na_2S_2O_3$ in 15 mL of saturated aqueous $NaHCO_3$). The combined organic layers were washed with brine and then dried over anhydrous magnesium sulfate before evaporation of the solvent in vacuo to give the crude product, which was purified by flash chromatography using a gradient of 0–50% ethyl acetate in hexanes to give compound **12** as a yellow oil. Yield: 116 mg, 49%. R_f = 0.75 (100% ethyl acetate). ^{1}H NMR ($CDCl_3$, 500 MHz) δ 9.65 (s, 1H), 7.46 (d, *J* = 8.7 Hz, 2H), 6.92 (d, *J* = 8.7 Hz, 2H), 6.85-6.77 (m, 1H), 5.87 (d, *J* = 15.0 Hz, 1H), 5.48 (s, 1H), 5.44 (s, 1H), 4.06 (s, 1H), 3.87-3.77 (m, 5H), 3.32 (m, 2H), 2.39 (dd, *J* = 14.3, 8.4 Hz, 1H), 2.31 (dd, *J* = 14.2, 7.6 Hz, 1H), 1.57-1.49 (m, 2H), 1.37-1.28 (m, 4H), 1.27 (s, 3H), 0.90 (t, *J* = 6.8 Hz, 3H); ^{13}C NMR ($CDCl_3$, 125 MHz) δ 202.4, 165.0, 160.4, 137.5, 130.0, 127.9, 127.5, 113.8, 101.5, 85.1, 76.0, 55.4, 39.6, 37.4, 36.7, 29.3, 29.1, 22.4, 18.1, 14.0; HRMS for $C_{22}H_{31}NO_5$ $[M-H]^-$ calcd. 388.2130, found 388.2126.

1.2.9. (4*R*,5*R*)-2-(4-Methoxyphenyl)-5-methyl-*N*-(3-oxo-3-(pentylamino)propyl)-5-((*E*)-4-oxo-4-(pentylamino)but-2-en-1-yl)-1,3-dioxane-4-carboxamide (15)

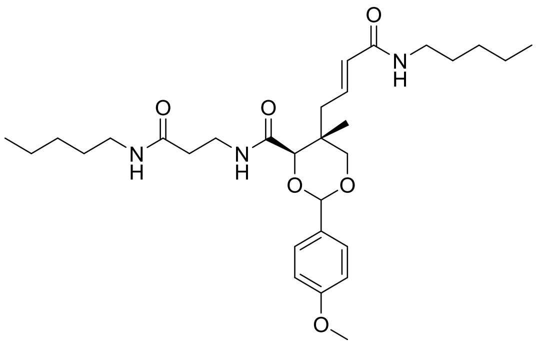

To a suspension of compound **12** (110 mg, 0.28 mmol) in a mixture of acetone and CH_2Cl_2 (3:1, v/v, 12 mL) was added a freshly prepared solution of NaH_2PO_4 (339 mg, 2.8 mmol) and $NaClO_2$ (160 mg, 1.4 mmol) in water (4 mL). The mixture was stirred at rt for 15 min. After evaporation of the solvent in vacuo, the residue was dissolved in a saturated solution of sodium sulfite (10 mL). The aqueous solution was extracted with ethyl acetate (3 × 25 mL). The combined organic layers were dried over magnesium sulfate and concentrated to give the crude acid **13** which was used without purification due to its poor stability.

To compound **14**, prepared with the method of Hoegl et al. [5], was added EDC (162 mg, 0.85 mmol), HOBt (137 mg, 1.0 mmol) and the flask was purged with nitrogen. A solution of the crude acid **13** in THF was added followed by DIPEA (0.49 mL, 2.8 mmol) and the mixture was stirred for 16 h at rt. The reaction mixture was poured into a separatory funnel containing an aqueous solution of saturated ammonium chloride, before extraction in ethyl acetate (3 × 40 mL). The combined organic layers were dried over magnesium sulfate and evaporated to give the crude product, which was purified by flash chromatography using a gradient of 0–100% ethyl acetate in hexanes, followed by 0–10% methanol in ethyl acetate to yield compound **15** as white gummy solid. Yield: 78 mg, 51%. R_f = 0.15 (100% ethyl acetate). ^{1}H NMR ($CDCl_3$,500 MHz) δ 7.40 (d, *J* = 8.7 Hz, 2H), 7.01 (s, 1H), 6.92 (d, *J* = 8.7 Hz, 2H), 6.86 (dt, *J* = 15.4, 7.9 Hz, 1H), 5.91 (d, *J* = 15.0 Hz, 1H), 5.86 (s, 1H), 5.45 (s, 1H), 5.42 (s, 1H), 4.20 (s, 1H), 3.86-3.75 (m, 5H), 3.62-3.47 (m, 2H), 3.31 (m, 2H), 3.20 (m, 2H), 2.54-2.34 (m, 4H), 1.55-1.42 (m, 4H), 1.37-1.26 (m, 8H), 1.14 (s, 3H), 0.89 (m, 6H);

^{13}C NMR ($CDCl_3$,125 MHz) δ 170.6, 169.3, 165.4, 160.3, 138.6, 129.9, 127.5, 127.5, 113.7, 101.3, 81.9, 76.1, 55.3, 39.6, 39.6, 37.9, 36.1, 35.9, 35.1, 29.3, 29.2, 29.1, 29.0, 22.3, 22.3, 17.9, 14.0, 14.0; HRMS for $C_{30}H_{47}N_3O_6$ $[M+H]^+$ calcd. 546.3538, found 546.3535.

1.2.10. (5*R*,6*R*,*E*)-6-Hydroxy-5-(hydroxymethyl)-5-methyl-*N*-7-(3-oxo-3-(pentylamino)propyl)- *N*-1-pentylhept-2-enediamide (16)

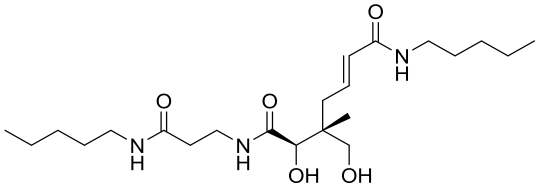

Acetic acid (3 mL, 90% in water) was added to compound **15** (70 mg, 0.13 mmol) in a 10 mL round-bottomed flask and the colorless mixture was stirred for 16 h at rt. The solvent was evaporated in vacuo by rotary evaporation and the residue was purified by flash chromatography using a gradient of 0–100% ethyl acetate in hexanes, followed by 0–25% methanol in ethyl acetate to yield compound **16** as a colorless oil. Yield: 46 mg, 84%. R_f = 0.33 (methanol/ethyl acetates, 1:9). ^{1}H NMR ($CDCl_3$,500 MHz) δ 7.56 (t, *J* = 6.1 Hz, 1H), 6.78 (dt, *J* = 15.1, 7.8 Hz, 1H), 6.64 (t, *J* = 5.4 Hz, 1H), 6.01 (t, *J* = 5.6 Hz, 1H), 5.84 (d, *J* = 15.1 Hz, 1H), 4.03 (s, 1H), 3.65-3.49 (m, 4H), 3.27 (m, 2H), 3.19 (m, 2H), 2.50-2.39 (m, 2H), 2.31 (d, *J* = 7.8 Hz, 2H), 1.56-1.44 (m, 4H), 1.36-1.27 (m, 8H), 0.99 (s, 3H), 0.89 (m, 6H); ^{13}C NMR ($CDCl_3$,125 MHz) δ 173.6, 171.4, 166.0, 140.4, 126.4, 76.0, 68.3, 42.6, 39.6, 39.5, 36.5, 35.6, 35.2, 29.1, 29.1, 29.0, 29.0, 22.2, 22.2, 19.5, 13.8, 13.8; HRMS for $C_{22}H_{41}N_3O_5$ $[M+H]^+$ calcd. 428.3119, found 428.3110.

2. Biological studies

2.1. General information

The reagents used to prepare buffer solutions and cell culture broths were obtained from Fisher Scientific (Whitby, ON, Canada), VWR (Mississauga, ON, Canada), and Sigma-Aldrich Canada (Oakville, ON, Canada). *E. coli* and *S. aureus* were grown in Difco^{TM} tryptic soy broth, purchased from BD (Franklin Lakes, NJ, United States). Expression plasmids for *E.coli* PanK were generously provided by Prof. Gerry Wright (McMaster University, Canada).

2.2. PanK enzyme activity

E. coli pantothenate kinase was expressed and purified as previously described [6]. The assay couples the production of ADP to the consumption of NADH through the activity of pyruvate kinase and lactic dehydrogenase. NADH consumption was monitored at 340 nm. Reactions were performed at 25 °C in a Molecular Devices SpectraMax® i3. Each reaction mixture (200 μL) contained ATP (62.5μM), pantothenate (25μM), NADH (0.3 mM), phosphor(enol)pyruvate (2 mM), $MgCl_2$ (10 mM), KCl (20 mM), pyruvate kinase/lactic dehydrogenase enzymes from rabbit muscle (10 μL), PanK (150 nM) and compound **16** (20–1000 μM) in Tris-HCl buffer (50 mM, pH 7.6). The reaction was initiated with the addition of ATP. All data are the average of the results from at least four separate experiments.

2.3. Antibacterial activity

Antimicrobial activity of compound **16** was tested against *S. aureus* ATCC 29213 and *E. coli* ATCC 25922, which were grown on Tryptic soy medium. The antibacterial activity was determined using the microdilution broth method in 96-well microtiter plates according to the National Committee for Clinical Laboratory Standards (NCCLS) [7]. For solubility purposes, compound **16** was dissolved in 50% (v/v) DMSO in water and then serially diluted to concentrations of 1, 2, 4, 8, 16, 32, 64, 128, 256, 512 μg/mL. A bacterial suspension of 10^6 cfu/mL was then used to inoculate each well, followed by incubation for 20 h at 37 °C. The MIC was taken as the lowest concentration demonstrating no visible growth in the broth. All data are the average of the results from at least four separate experiments.

2.4. Antiplasmodial activity

The in vitro *P. falciparum* growth assay was performed as previously reported [8]. All malaria parasite experiments were performed using the 3D7 strain of *P. falciparum*.

3. References

(1) Akinnusi, T. O.; Vong, K.; Auclair, K. *Bioorg. Med. Chem.* **2001**, 19, 2696-2706.

(2) Guindon, Y.; Yoakim, C.; Gorys, V.; Ogilvie, W. W.; Delorme, D.; Renaud, J.; Robinson, G.; Lavallee, J. F.; Slassi, A.; Jung, G.; Rancourt, J. *J. Org. Chem.* **1994**, 59, 1166-1178.

(3) Pardin, C.; Gillet, S. M.; Keillor, J. W. *Bioorganic & medicinal chemistry* **2006**, 14, 8379-8385.

(4) Masanori, K.; Eiichi, S.; Koichi, T.; Akira, I. Jpn. Kokai Tokkyo Koho. JP 02045456, Feb 15, 1990.

(5) Hoegl, A.; Darabi, H.; Tran, E.; Awuah, E.; Kerdo, E. S.; Habib, E.; Saliba, K. J.; Auclair, K. *Bioorg Med Chem Lett* **2014,** *24*, 3274-3277.

(6) Nazi, I.; Koteva, K. P.; Wright, G. D. *Anal. Biochem*. **2004**, 324, 100-105.

(7) NCCLS—National Committee for Clinical Laboratory Standards: Performance Standards for Antimicrobial Susceptibility Testing. 14th ed. Approved Standard, document M100-S14, NCCLS, Wayne, Pennsylvania; 2004.

(8) de Villiers, M.; Macuamule, C.; Spry, C.; Hyun, Y. M.; Strauss, E.; Saliba, K. J. *ACS Med. Chem. Lett*. **2013**, 4, 784-789.
