# Supplementary material for: A cross-metathesis approach to novel pantothenamide derivatives
Source: Beilstein J Org Chem. 2016 May 13;12:963–8. doi: 10.3762/bjoc.12.95 (PMC4902030; doi:10.3762/bjoc.12.95)

Supporting Information File 2

for

A cross-metathesis approach to novel pantothenamide derivatives

Jinming Guan[1], Matthew Hachey[1], Lekha Puri[1], Vanessa Howieson[2], Kevin J. Saliba[2,3] and Karine Auclair*[1]

Address: [1]Department of Chemistry, McGill University, 801 Sherbrooke Street West, Montreal, H3A 0B8, Canada, [2]Research School of Biology, College of Medicine, Biology and Environment, The Australian National University, Canberra, Australian Capital Territory, 2601, Australia and [3]Medical School, College of Medicine, Biology and Environment, The Australian National University, Canberra, Australian Capital Territory, 2601, Australia

Email: Karine Auclair - karine.auclair@mcgill.ca

* Corresponding author

NMR spectra

Content

NMR spectra for compounds **6**, **7**, **9**, **10d**, **11d**, **12**, **15**, and **16**.

^{1}H and ^{13}C NMR spectra for 6

500 MHz ^{1}H NMR spectrum of compound 6 ($CDCl_3$)

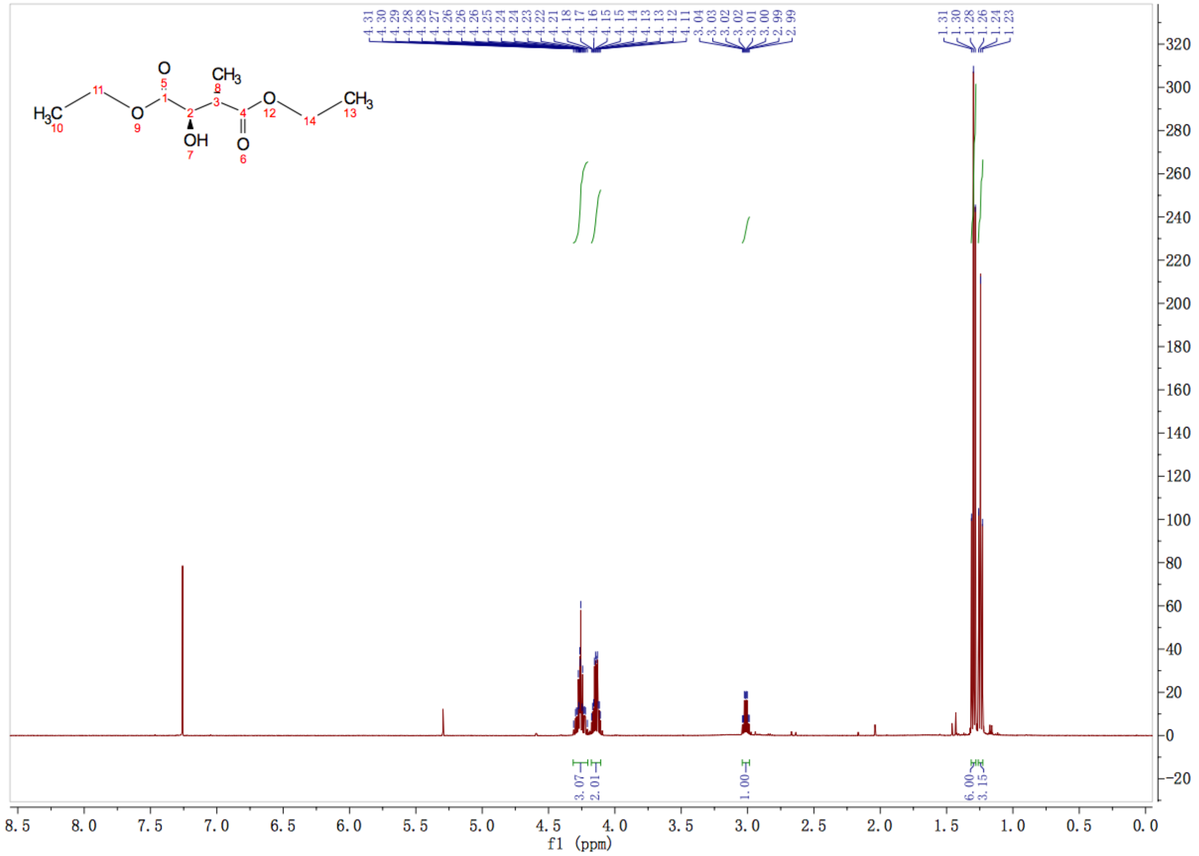

125 MHz ^{13}C NMR Spectrum of Compound 6 ($CDCl_3$)

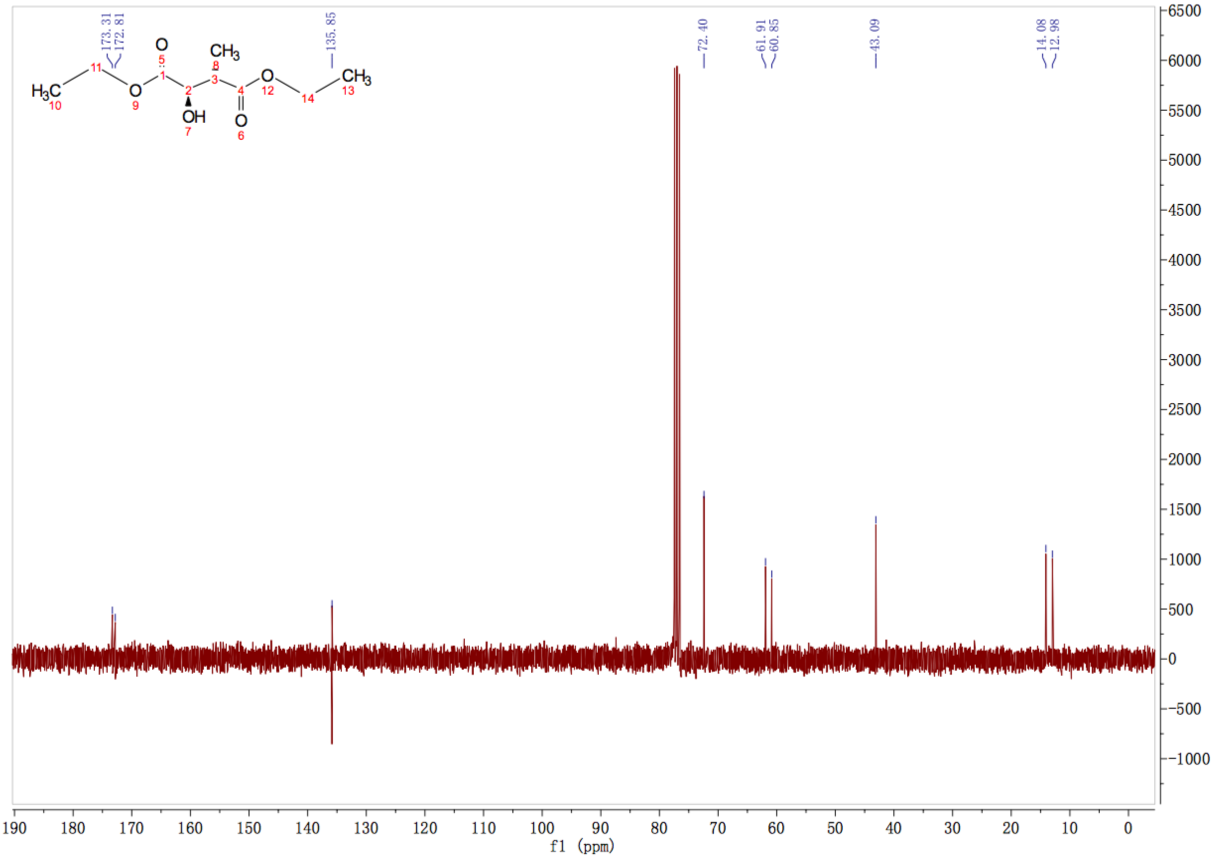

^{1}H and ^{13}C NMR spectra for 7

500 MHz ^{1}H NMR spectrum of compound 7 ($CDCl_3$)

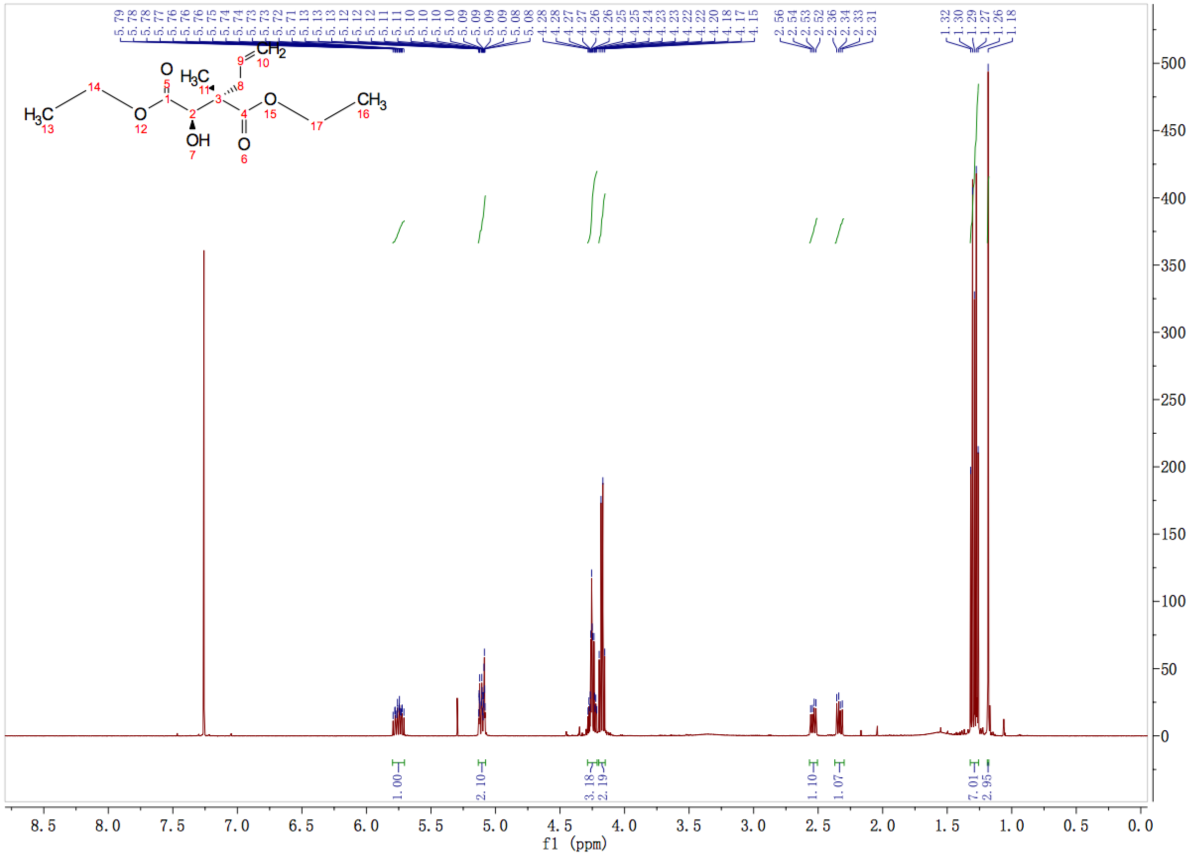

125 MHz ^{13}C NMR spectrum of compound 7 ($CDCl_3$)

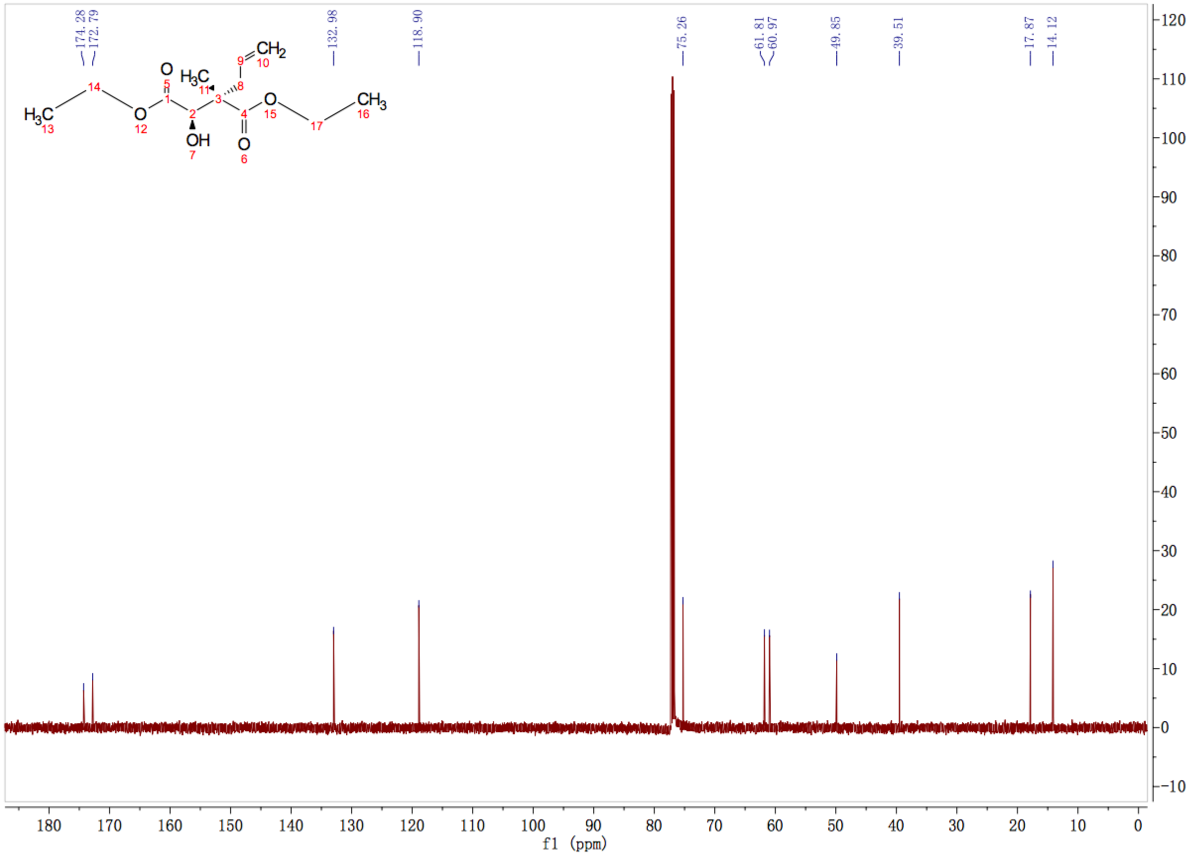

^{1}H and ^{13}C NMR spectra for 9

500 MHz ^{1}H NMR spectrum of compound 9 ($CDCl_3$)

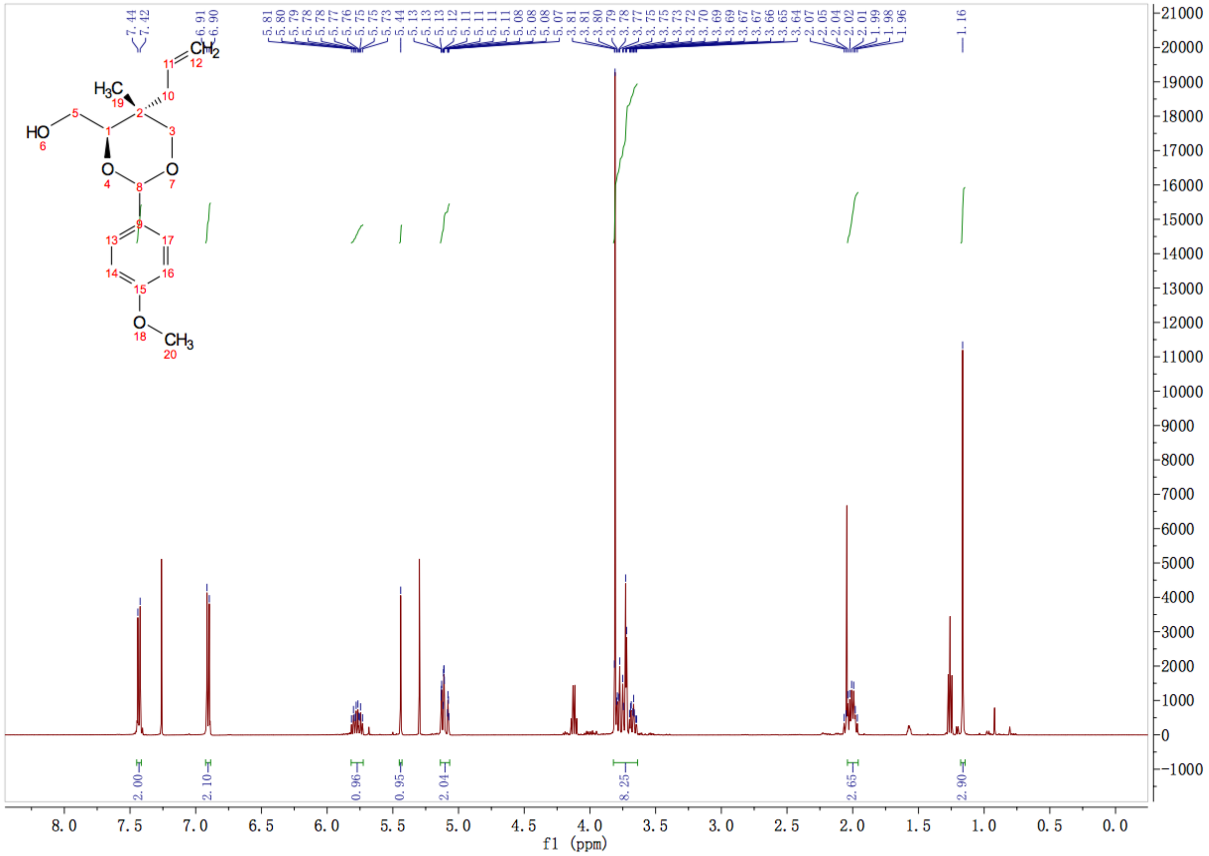

125 MHz ^{13}C NMR Spectrum of Compound 9 ($CDCl_3$)

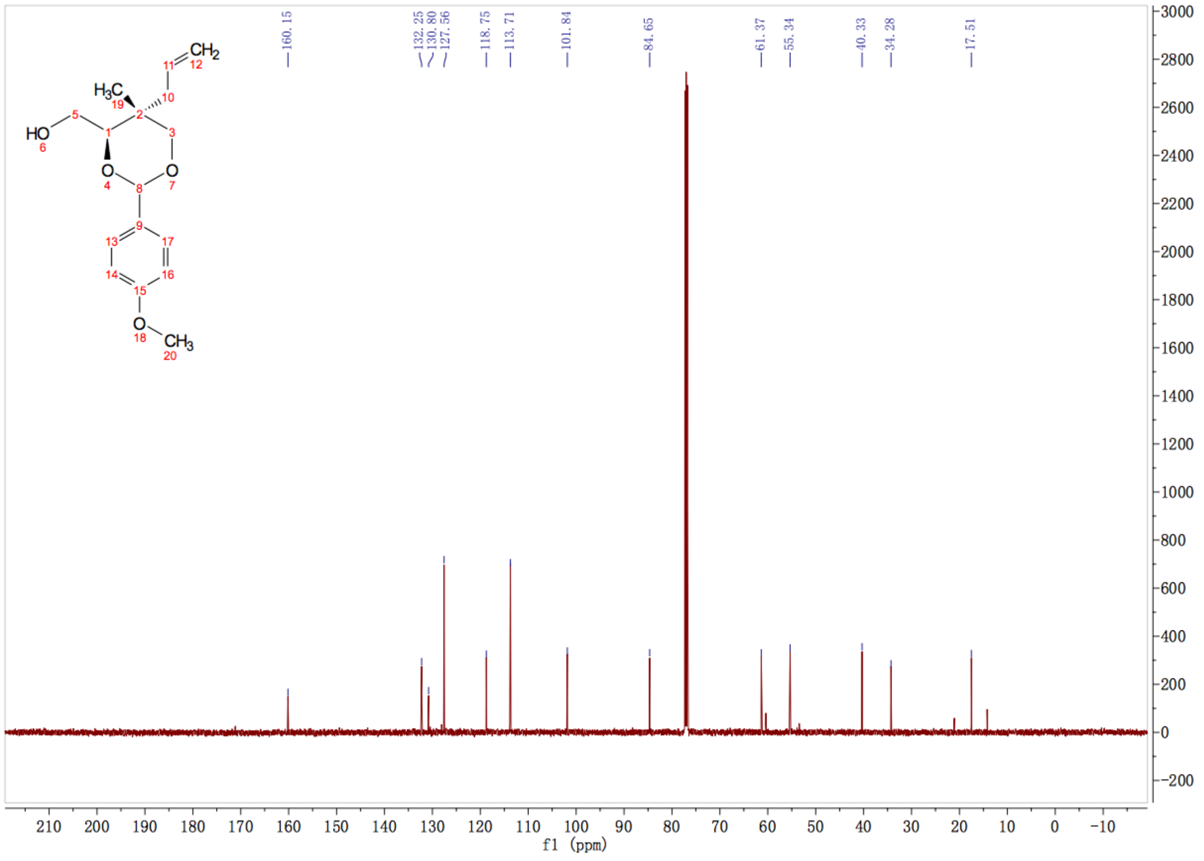

^{1}H and ^{13}C NMR spectra for 10d

300 MHz ^{1}H NMR spectrum of compound 10d ($CDCl_3$)

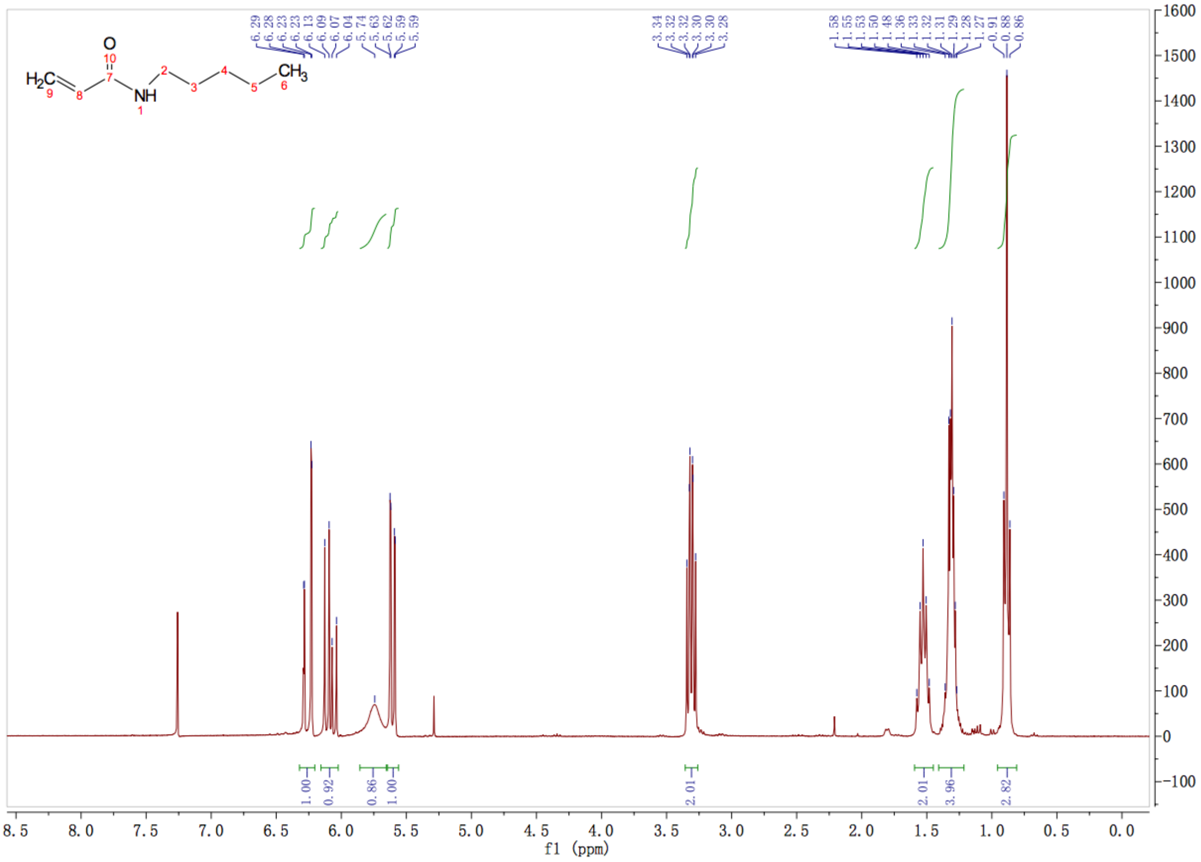

125 MHz ^{13}C NMR Spectrum of Compound 10d ($CDCl_3$)

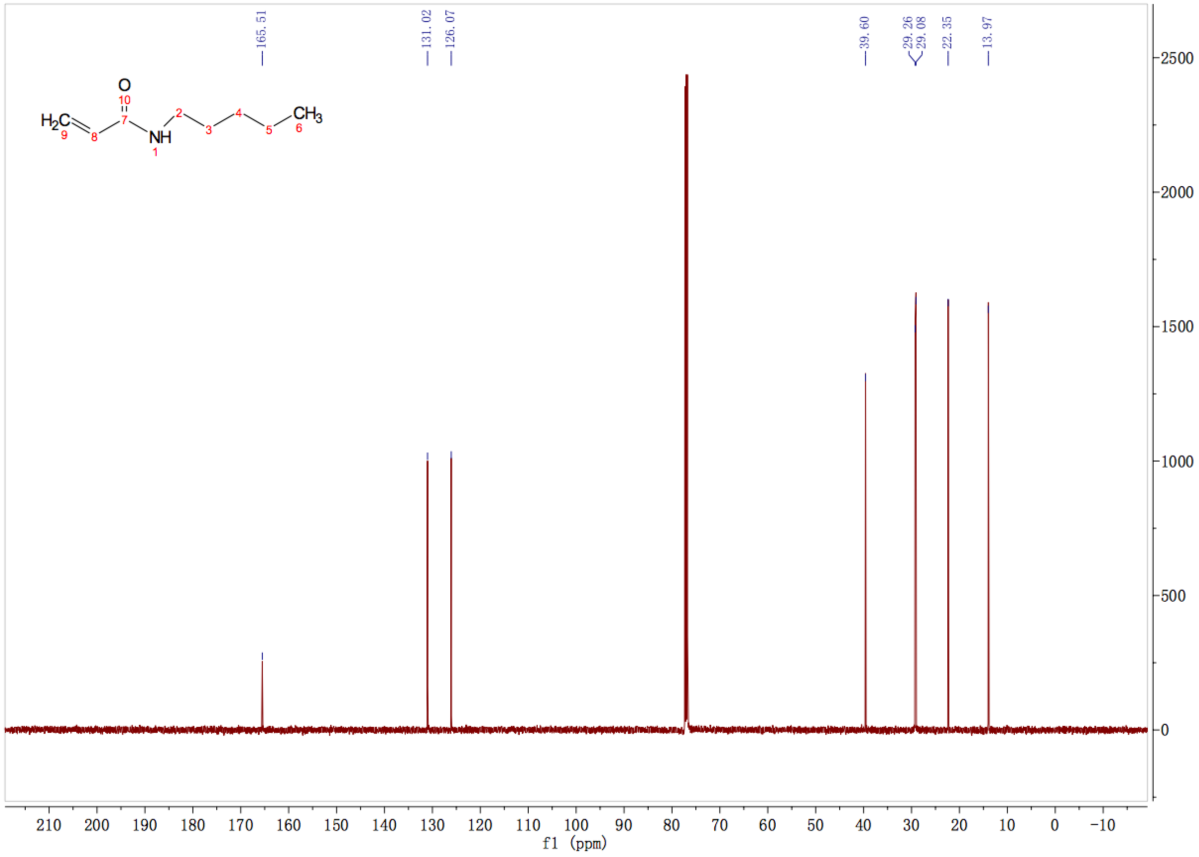

^{1}H and ^{13}C NMR spectra for 11d

500 MHz ^{1}H NMR spectrum of compound 11d ($CDCl_3$)

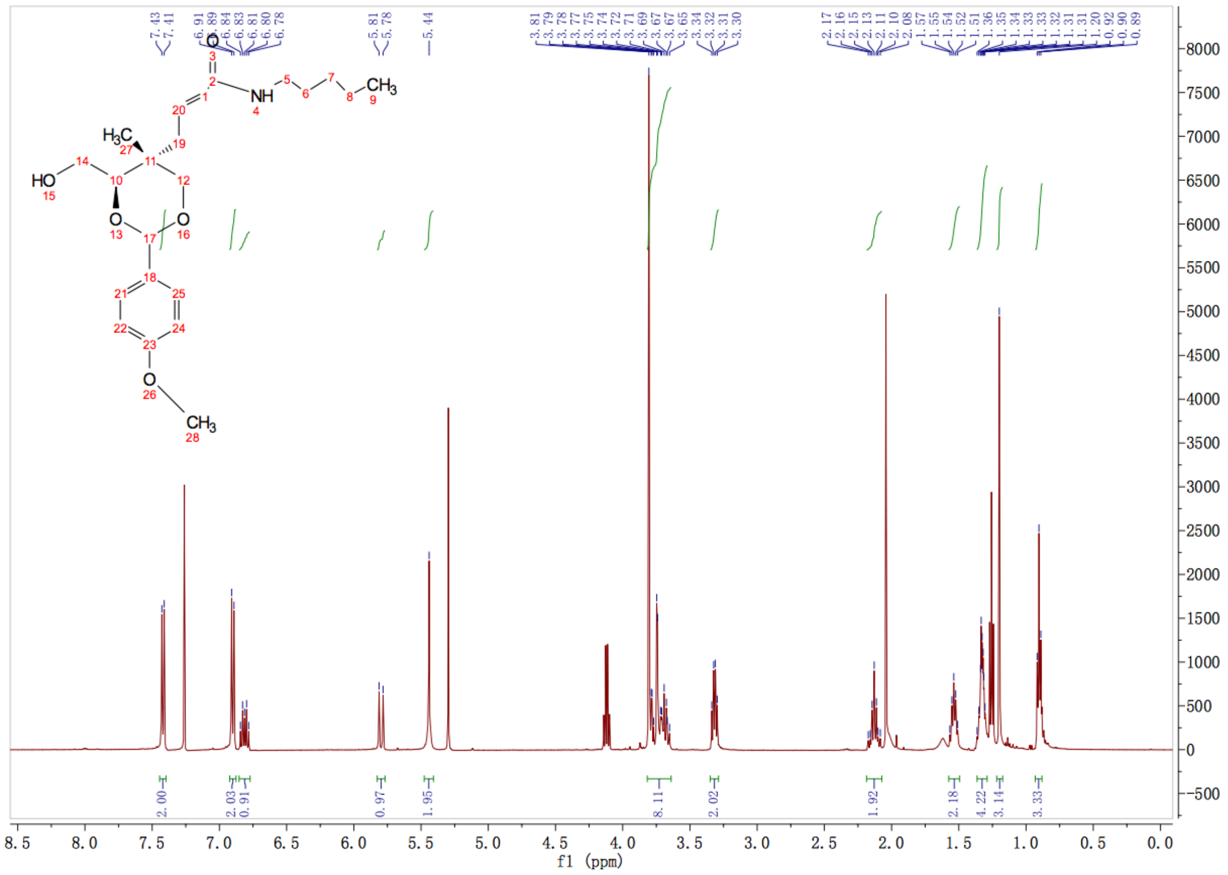

125 MHz ^{13}C NMR spectrum of compound 11d ($CDCl_3$)

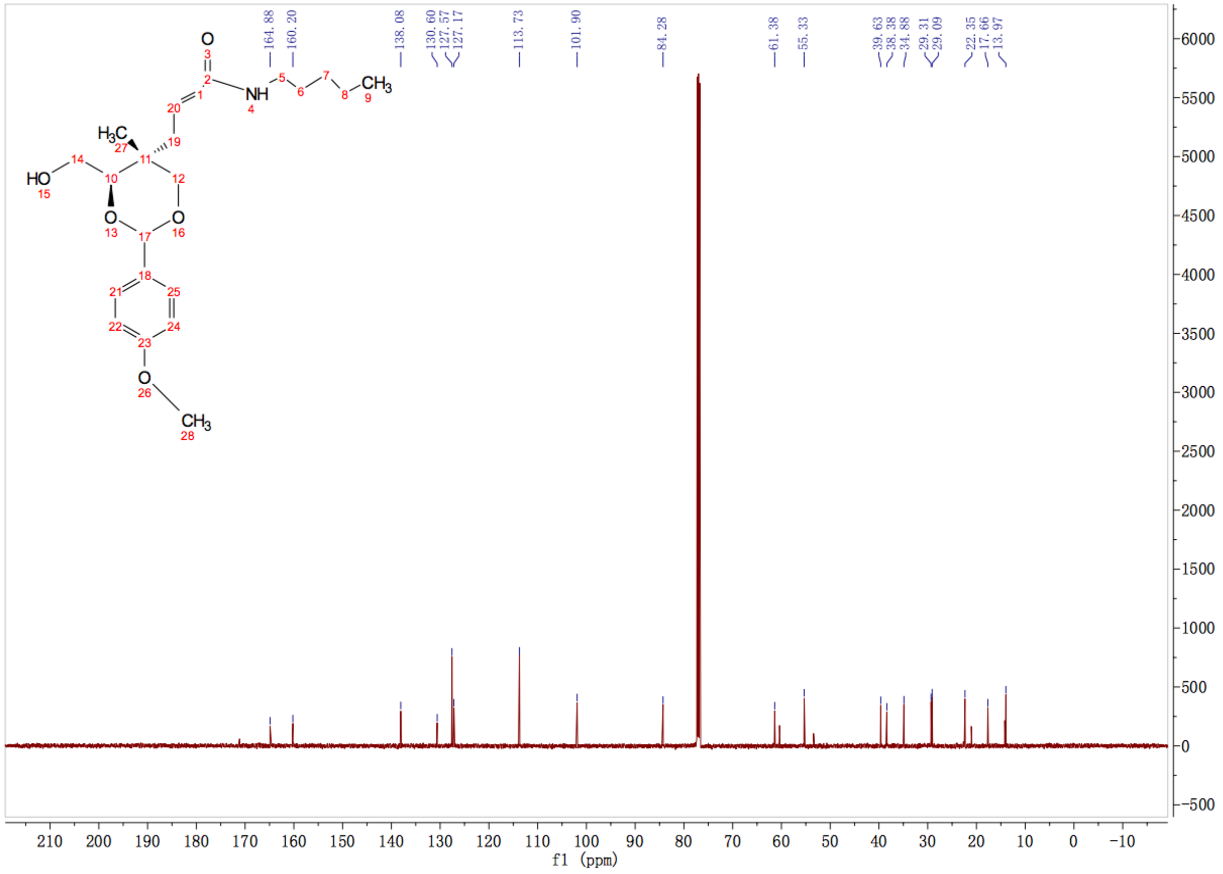

^{1}H and ^{13}C NMR spectra for 12

500 MHz ^{1}H NMR spectrum of compound 12 ($CDCl_3$)

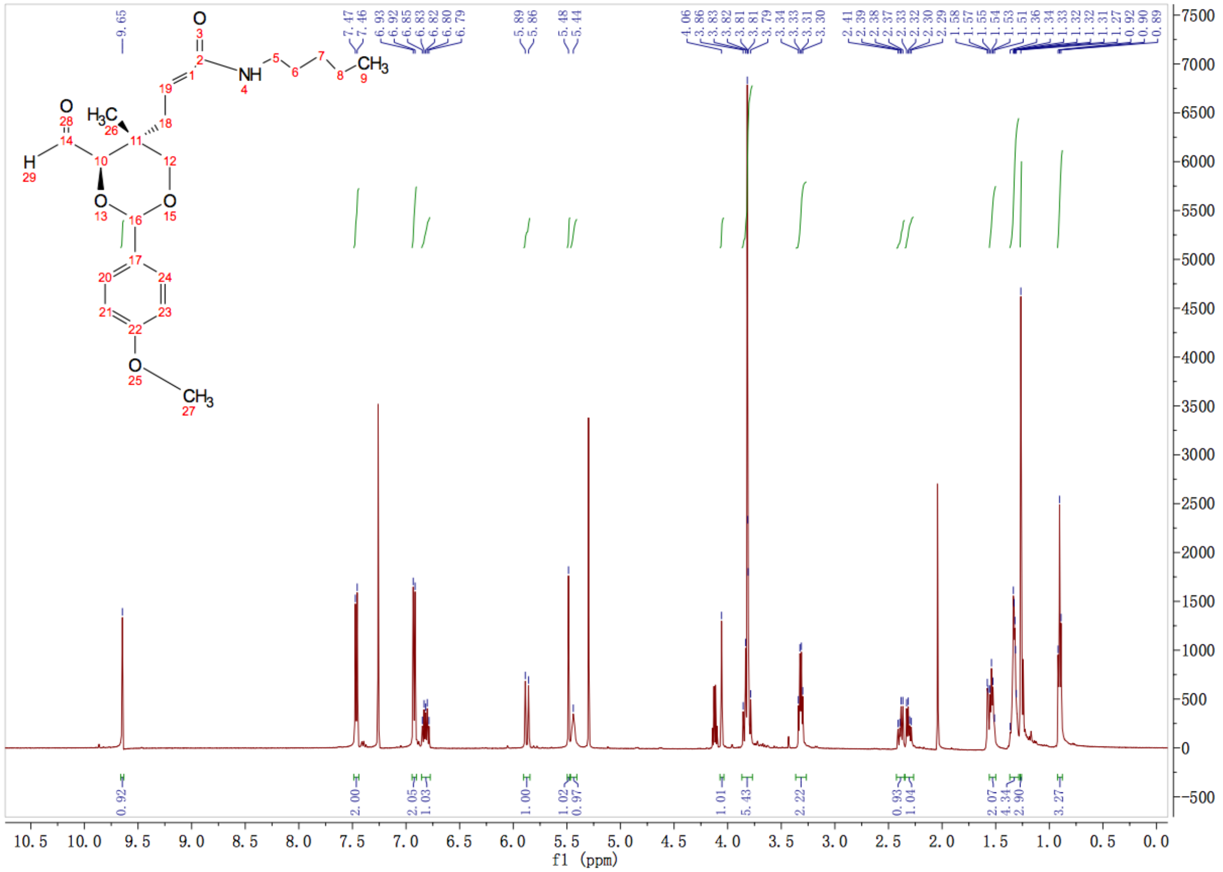

125 MHz ^{13}C NMR spectrum of compound 12 ($CDCl_3$)

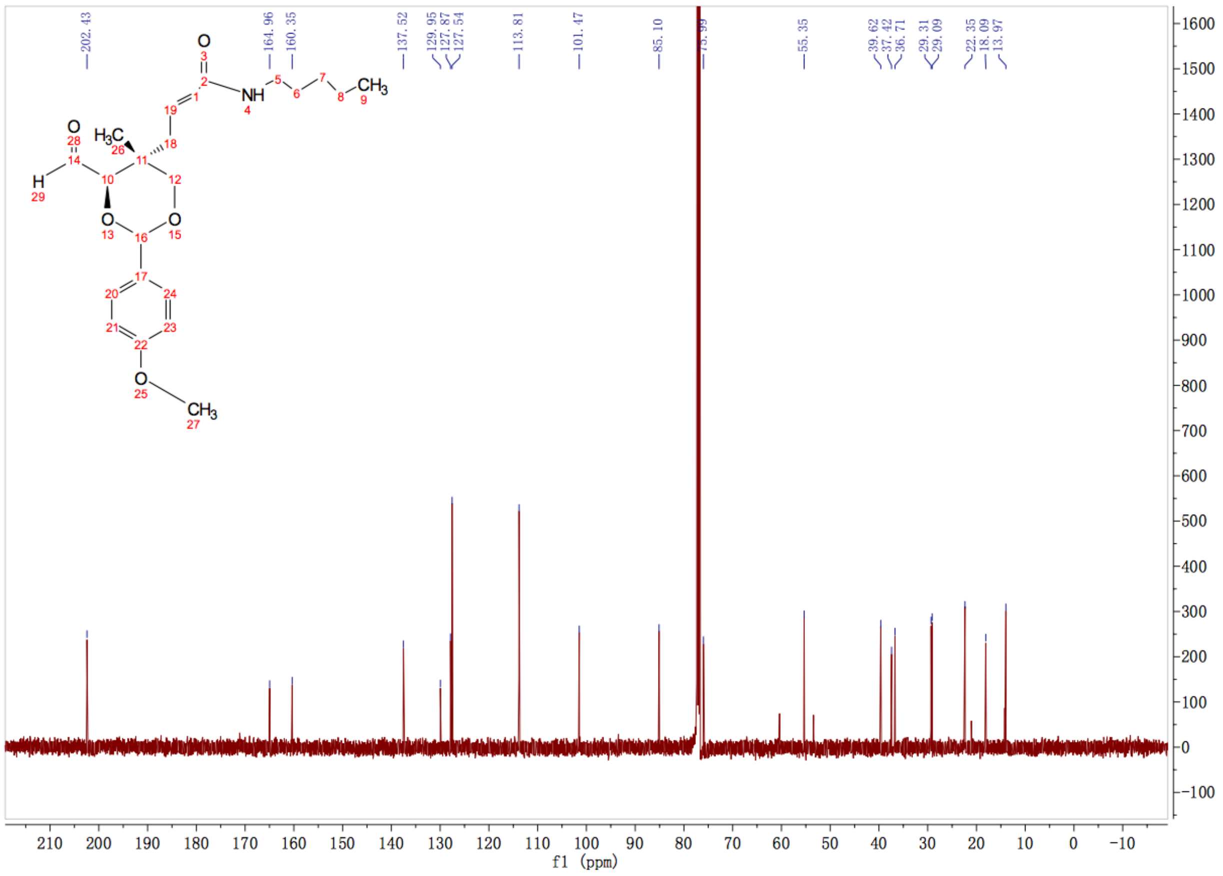

^{1}H and ^{13}C NMR spectra for 15

500 MHz ^{1}H NMR spectrum of compound 15 ($CDCl_3$)

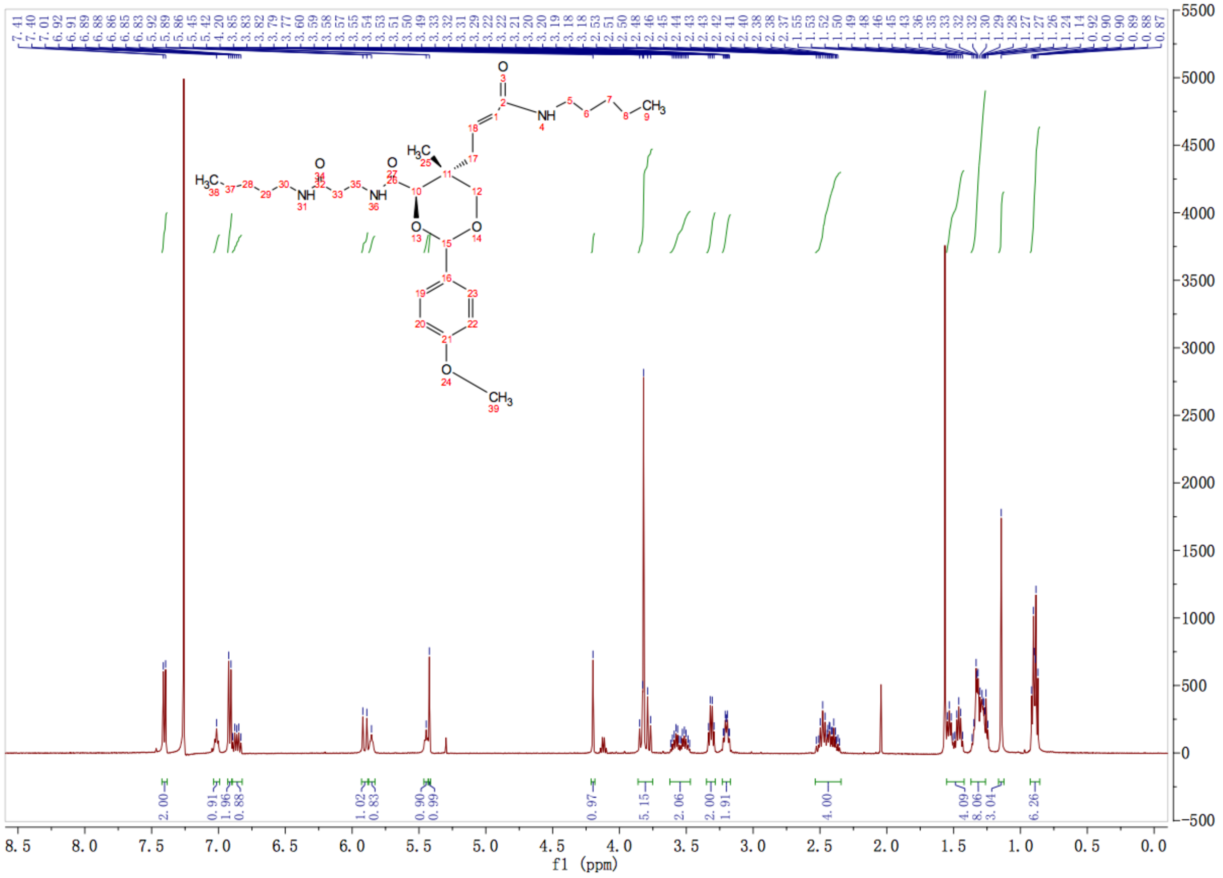

125 MHz ^{13}C NMR spectrum of compound 15 ($CDCl_3$)

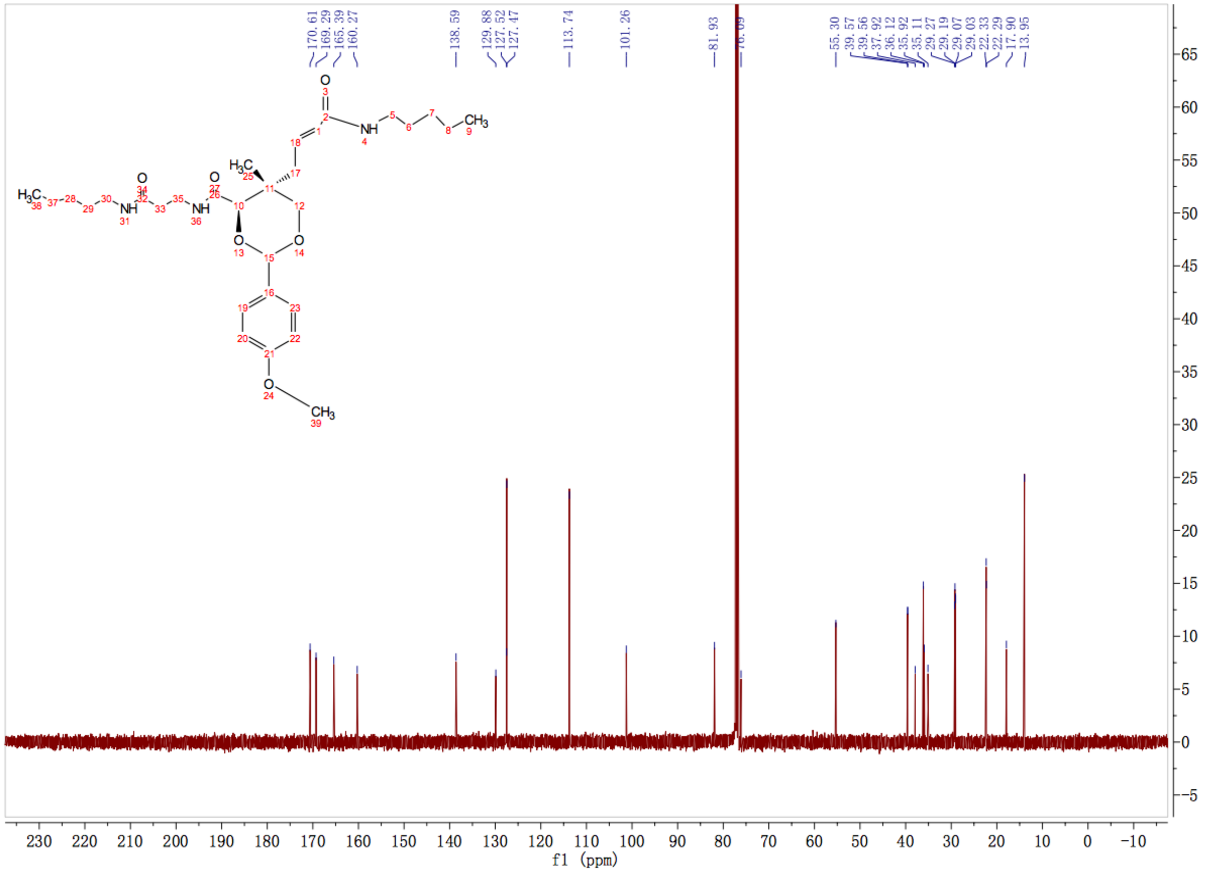

500 MHz ^{1}H-^{13}C HSQC NMR spectrum of compound 15 ($CDCl_3$)

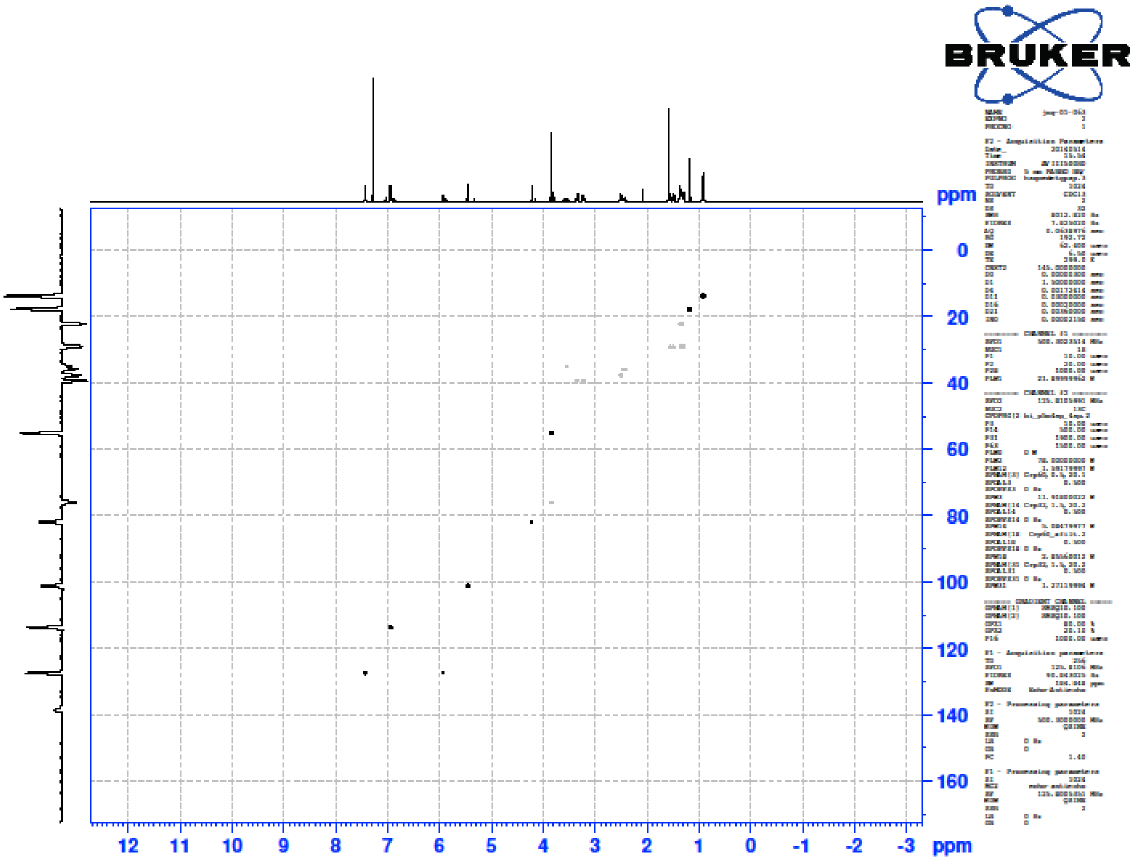

^{1}H and ^{13}C NMR spectra for 16

500 MHz ^{1}H NMR spectrum of compound 16 ($CDCl_3$)

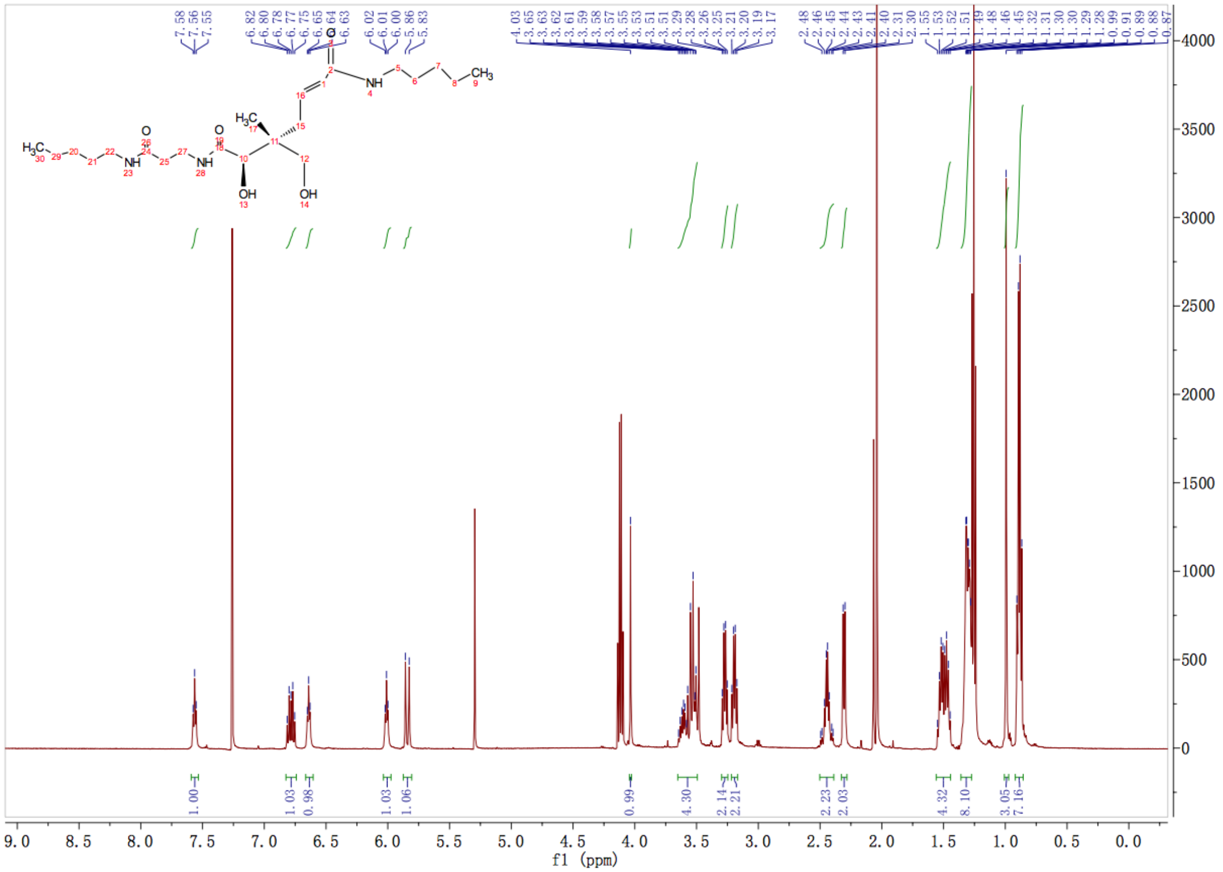

125 MHz ^{13}C NMR spectrum of compound 16 ($CDCl_3$)

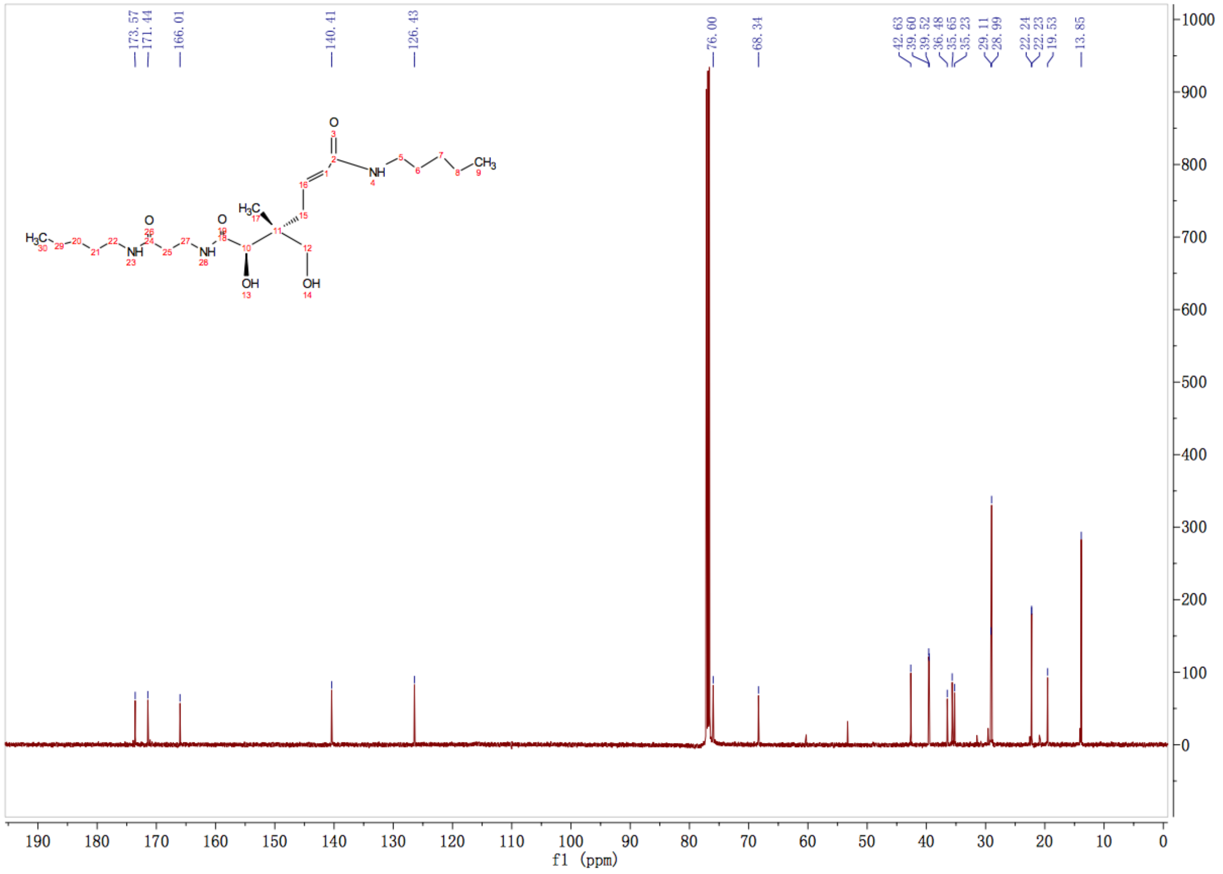

500 MHz ^{1}H-^{13}C HSQC NMR spectrum of compound 16 ($CDCl_3$)

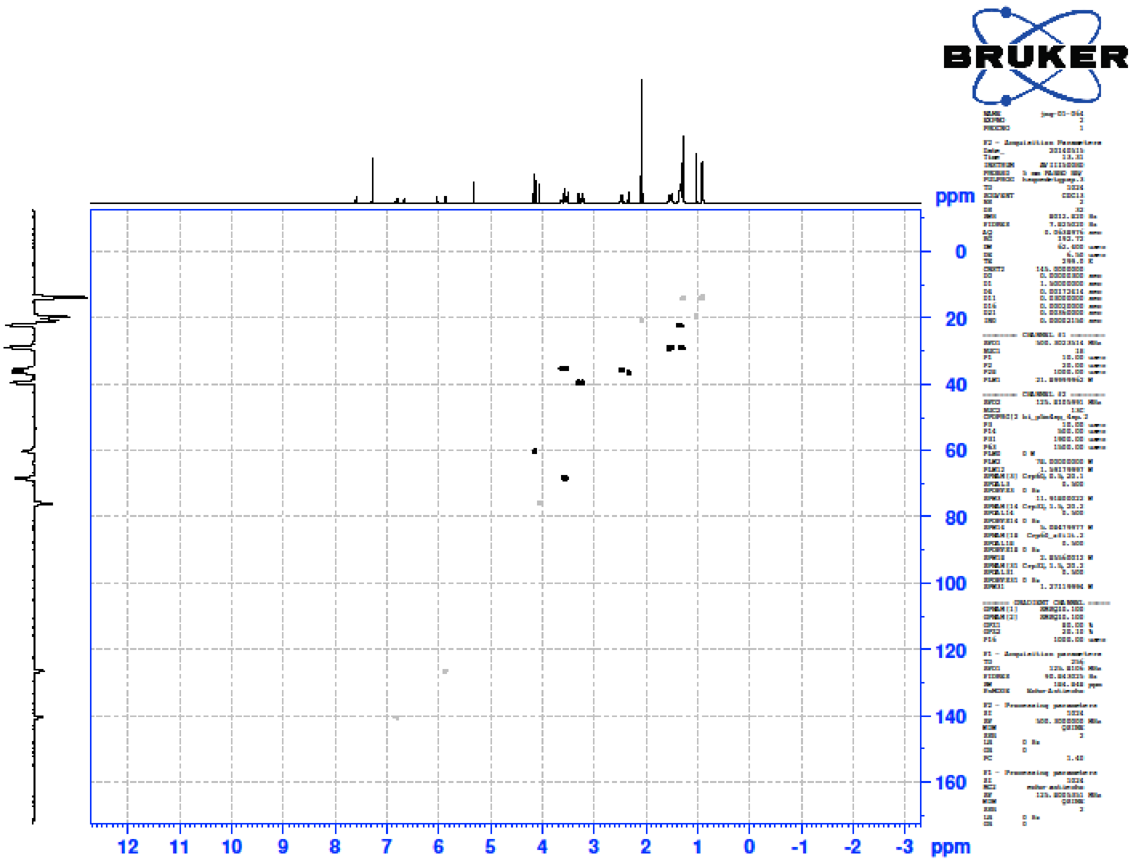

Supplement: File 2 — NMR spectra. [file Beilstein_J_Org_Chem-12-963-s002.pdf]
